# Statistical and Machine Learning-Driven Optimization of Mechanical Properties in Designing Durable HDPE Nanobiocomposites

**DOI:** 10.3390/polym13183100

**Published:** 2021-09-15

**Authors:** Anusha Mairpady, Abdel-Hamid I. Mourad, Mohammad Sayem Mozumder

**Affiliations:** 1 Chemical and Petroleum Engineering Department, UAE University, Al Ain 15551, United Arab Emirates; mairpadyanu@uaeu.ac.ae; 2 Mechanical Engineering Department, UAE University, Al Ain 15551, United Arab Emirates; ahmourad@uaeu.ac.ae; 3 National Water and Energy Center, United Arab Emirates University, Al Ain 15551, United Arab Emirates; 4 Mechanical Design Department, Faculty of Engineering, Helwan University, Cairo 11795, Egypt

**Keywords:** polymer–matrix composites (PMC), computational modeling, statistical optimization, mechanical properties, durability, artificial neural network (ANN), genetic algorithm (GA)

## Abstract

The selection of nanofillers and compatibilizing agents, and their size and concentration, are always considered to be crucial in the design of durable nanobiocomposites with maximized mechanical properties (i.e., fracture strength (FS), yield strength (YS), Young’s modulus (YM), etc). Therefore, the statistical optimization of the key design factors has become extremely important to minimize the experimental runs and the cost involved. In this study, both statistical (i.e., analysis of variance (ANOVA) and response surface methodology (RSM)) and machine learning techniques (i.e., artificial intelligence-based techniques (i.e., artificial neural network (ANN) and genetic algorithm (GA)) were used to optimize the concentrations of nanofillers and compatibilizing agents of the injection-molded HDPE nanocomposites. Initially, through ANOVA, the concentrations of TiO_2_ and cellulose nanocrystals (CNCs) and their combinations were found to be the major factors in improving the durability of the HDPE nanocomposites. Further, the data were modeled and predicted using RSM, ANN, and their combination with a genetic algorithm (i.e., RSM-GA and ANN-GA). Later, to minimize the risk of local optimization, an ANN-GA hybrid technique was implemented in this study to optimize multiple responses, to develop the nonlinear relationship between the factors (i.e., the concentration of TiO_2_ and CNCs) and responses (i.e., FS, YS, and YM), with minimum error and with regression values above 95%.

## Highlights

HDPE nanocomposites were developed using injection molding.

The impact of the type and size of the nanofillers and the compatibilizing agent on the mechanical properties (i.e., fracture strength, yield strength, and Young’s modulus, etc.) of the developed nanocomposites was analyzed.

Both statistical (i.e., ANOVA, RSM) and machine learning techniques (i.e., ANN, GA, ANN-GA) were used to assess the effect of nanofillers and the compatibilizing agent on the durability of the developed HDPE nanobiocomposites.

Nano-TiO_2_ and cellulose nanocrystals were found to have a positive impact on improving the mechanical integrity of the HDPE nanobiocomposites.

Artificial neural network with genetic algorithm (ANN-GA) provided a better prediction with minimum error than the response surface methodology.

## 1. Introduction

The success of the designed biomaterials to replace natural scaffolds depends on their ability to facilitate cell growth. More specifically, they should aid the cross-talk between cells, growth factors, and protein, thereby initiating cell adhesion, proliferation, and differentiation [1,2]. For these procedures to occur in the proper sequence, an extracellular matrix having the required mechanical integrity (i.e., durability) and biocompatibility is much needed [3]. Over the last few decades, polymers have proven to be excellent candidates, exhibiting the properties required for materials to be in the extracellular matrices. Polyurethane [4], poly(lactic acid) [5,6], polyester [7,8], and polyethylene [9,10] are among the most commonly used polymers in tissue engineering. In this study, high-density polyethylene (HDPE) was considered the base polymer due to its long-reported success in tissue engineering. To be more specific, HDPE is an organic polymer and possesses good mechanical and physical properties, excellent chemical resistance, and biocompatibility, which make it suitable for various biological applications [11].

Earlier, metal oxides, carbon nanotubes, natural fibers, etc. were being added to the polymer as fillers to reduce the production cost of polymeric materials. Over time, these fillers, particularly nanofillers, have become an inseparable part of polymer composites [12]. The application of nanofillers is not only limited to improving polymers’ mechanical properties, but also to enhance their conductivity, biocompatibility, thermal stability, and so on. In simple words, fillers should provide the properties, which are inherently absent in the polymer, without changing its intrinsic features, or adding extra weight, or affecting its processability [12,13]. Among the inorganic nanofillers, titanium dioxide has proven to be an excellent candidate to be combined with HDPE, owing to its recommendable mechanical properties, thermal degradation, reduction of ultraviolet (UV) light, and resistance to corrosion [14]. The combination of HDPE and titanium dioxide has shown some promising results in tissue engineering [9,15,16].

In the past few decades, CNCs have gained much interest owing to their strong physical, mechanical property, nanoscale dimension, high surface area, and low density. These biopolymers can easily be modified to be biodegradable and renewable [15,16]. More specifically, CNCs individually has a young’s modulus of 167.5 GPa and impressive mechanical strength [17]. Due to these attributes, this study also considers the impact of incorporating CNCs into the polymer mix as one of the design parameters. Moreover, n-TiO_2_ and n-CNCs have been proven to improve the cell adhesion and proliferation of chondrocytes and to demonstrate antimicrobial properties [9,15,18,19,20].

However, the binding of polar nanoparticles to the non-polar polymer matrix remains a critical concern in nanocomposites design. Typically, compatibilizers or coupling agents are added to the composites to modify the composites’ interfaces. These agents may include saline [21,22], itaconic acid [23], and graft copolymers of maleic anhydride [24,25]. In this study, two different coupling agents, such as polyethylene grafted maleic anhydride and 3-(Trimethoxysilyl) propyl methacrylate, have been considered. In polyethylene grafted maleic anhydride, the maleic anhydride can be bonded with the metal oxides, e.g., TiO_2_, SiO_2,_ etc., while the polyethylene part of the coupling agent would have a physical interaction with the polyethylene composite matrix [26,27]. Typically, the nanoparticles have a high surface-to-volume ratio, attributed to a significant amount of surface energy and Vander Walls forces. These energies attract the nanoparticles to each other leading to the formation of aggregates onto the polymeric surfaces. On the other hand, saline coupling agents such as 3-(Trimethoxysilyl) propyl methacrylate increase the surface area and ensure a uniform distribution of the filler on the polymeric surfaces [21,28]. The mechanism of the compatibilizing agents’ binding to polyethylene is postulated and illustrated in Supplementary Figure S1 (polyethylene grafted maleic anhydride) and Figure S2 (3-(Trimethoxysilyl) propyl methacrylate).

The selection of the nanofillers and compatibilizers, their size, and their concentration vary significantly depending on the sought properties of the biomaterials and their potential application(s). It involves vigorous experimentation to tune up these parameters. However, the experimental runs required and the time duration needed can be reduced using statistical analyses and computational techniques [9,29]. Recently, multivariate statistical analyses have been popularly adopted, through which researchers can identify the optimal combination of the design factors and their interaction Typically, the research design involves the following steps: (1) determining the mathematical model coefficient from the relationship between the factors (i.e., independent variables) and the responses (i.e., dependent variables), (2) estimation of the predicted response, and (3) evaluation of the adequacy of the model. There are different techniques for multivariate analyses ranging from simpler ones such as factorial design to many complex ones such as the response of surface methodology (RSM) [30,31]. Box and Wilson were the first to develop RSM [32] to evaluate the interactions of various variables together and to specify an empirical equation depicting the impact of the variables and their interaction to the measured response(s). RSM has been used to optimize the fabrication method(s) of polymer nanocomposites [33,34], to evaluate the influence of numerous process variables on the technique(s) under consideration [34,35], and to quantify the effect of several different parameters on the properties of polymer nanocomposites [36,37]. However, only a linear relationship between the factors and the responses can be developed using RSM. Indeed, the nonlinear relationship with a complex situation is beyond the scope of the RSM method [30,31].

However, most of the practical problems deal with the nonlinear relationship between the dependent and independent variables [38]. Hence, artificial intelligence and machine learning have garnered significant interest in modeling the experimental data, as these models can develop and compute nonlinear relationships by exploiting the available experimental data [39,40]. An established model can significantly reduce the routine testing involved in developing new polymer composites. The techniques of machine learning include artificial neural network (ANN), genetic algorithm, support vector regression (SVR), etc. [41,42]. ANN can model complex data without the need for extensive experimental data [43,44]. This benefit can be explored to model and predict the properties of the polymer nanocomposites using ANN [45,46]. Although ANN establishes nonlinear relationships, it can be time-consuming and can lead to false convergence or enables local convergence, and overfitting [41,42]. However, these error(s) can be minimized using a genetic algorithm (GA) [47]. GA is based on the evolutionary computational technique using the principle of the survival of the fittest or natural selection [44,48]. In this method, [49] the undesired overfitting can be minimized by splitting the data into training and testing sets, and by dealing with higher population size [50]. Owing to these benefits, several studies have implemented the genetic algorithm to develop novel polymer nanocomposites [51,52].

To the best of our knowledge, the detailed and systematic approach using both statistical and machine learning techniques has not yet been implemented to determine the influence of the nanofiller(s) and compatibilizing agent on the mechanical integrity of HDPE nanocomposites. In this study, HDPE-nanofillers-compatibilizing agents nanocomposites were fabricated and characterized. The experimental dataset was then exposed to a single or combination of several different statistical and computational techniques to reduce the predicted error(s) and to generate the best model by using the experimental findings. A preliminary analyss of these results has been reported in [10]. The initial objective of this study is to eliminate the factors that have no influence or negative impact on the HDPE nanocomposites’ properties. Then, this study aims to develop a relationship between the factors (i.e., size and concentration of TiO_2_, the concentration of CNC, the effect of coupling agents, etc.) and responses (i.e., fracture strength (FS), yield strength (YS), Young’s modulus (YM) of the developed nanobiocomposites) to enhance the durability of the HDPE nanobiocomposites using nanofillers and coupling agents. TiO_2_ and CNCs nanofillers and polyethylene grafted maleic anhydride and 3(Trimethoxysilyl) propyl methacrylate coupling agents are considered as the factors. The final objective of this work is to find the best synergy of the ingredient(s) to improve the mechanical properties of HDPE nanobiocomposites by using ANOVA, RSM, ANN, and genetic algorithm (GA). However, the risk of undesirable local optimization in this regard is grossly overlooked in the literature. In this study, ANN-GA approach has been implemented to minimize the chances of local optimization.

## 2. Methodology

### 2.1. Materials

High-density polyethylene (HDPE) was used as the polymer matrix (having a melt flow index of 2.2 g/10 min at 123 °C). The two compatibilizing agents used in this study were polyethylene grafted maleic anhydride (PEgMAH) (having a viscosity of 500 Cp at 140 °C), 3-(Trimethoxysilyl) Propyl Methacrylate (MPS) (1.045 g/mL at 25 °C and boiling point at 190 °C). TiO_2_ nanoparticles (average particle size 150 nm, 21 nm) and cellulose nanocrystals (CNCs) were used as nanofillers. CNCs were obtained from the University of Maine (freeze-dried powder form and obtained from acid hydrolysis). These nanocrystals were 150–200 nm long and had a density of 1.5 g/cm^3^ when they are in dried powder form. A few other miscellaneous items used in this study were ethanol (with a vapor density of 1.59 and 99.8% pure) and ammonia (≥99.95% pure and boiling point at −33 °C). All the materials mentioned above were purchased from Sigma Aldrich (Munich, Germany) unless otherwise stated.

### 2.2. Design, Development, and Characterization of the HDPE Nanocomposites

#### 2.2.1. Functionalization of TiO_2_ Nanoparticles with MPS Coupling Agent

First, ten gm of n-TiO_2_ were uniformly distributed in 250 mL of ethanol by using a magnetic stirrer (Sigma Aldrich, Munich, Germany). Then, thirteen gm of ion-exchanged distilled water, 6.8 gm of ammonia, and 6.2 gm of MPS were added to the evenly homogenized M-(n-TiO_2_)-methanol mixture. The final mixture was ultra-sonicated for one hour, followed by mechanical stirring for 24 h. The free MPS was then removed from the mixture by flushing with water and ammonia for five sedimentation cycles. The samples were then dried at room temperature to obtain the functionalized/modified n-TiO_2_ (M-(n-TiO_2_)).

#### 2.2.2. Development of HDPE Nanocomposites

The HDPE nanocomposites were developed using a Ray-ran injection molding machine (Ray-Ran Test Equipment, Ltd., Warwickshire, UK) The randomized sample design was obtained using full factorial design and center composite design. Five factors (i.e., n-TiO_2_, n-CNC, PEgMAH, M-(n-TiO_2_), size of the nanofillers) with three replicates were used in a full factorial design, whereas two factors (i.e., n-TiO_2_ and n-CNC) with three center points and three replicates were applied in the center composite design for further modeling. The maximum concentrations of nanofillers and compatibilizing agents used in this study are 5 wt.%. Based on the sample design, the predetermined concentration of the factors and HDPE were blended utilizing the ultra-centrifugal mixture ((Retsch GmbH, ZM 200, Haan, Germany)The homogeneous formulation was then transferred to the barrel of the injection molding machine through the hopper. Our previous studies concluded that a barrel temperature of 150 °C for the residence time of 50 min is the optimum for processing HDPE [18]. After the retention time was elapsed, samples were injected into molds of desired shape using air pressure. The operating conditions used in the injection molding machine are presented in Table 1.

#### 2.2.3. Mechanical Testing

Briefly, 20 mm long dog-bone samples have been used to evaluate their mechanical durability using MTS universal testing machine (MTS system corporation, Eden Prairie, MN, USA). Fracture testing was carried out in a universal testing machine equipped with a 100 KN load cell, and samples were stretched with an overhead speed of 5 mm/min. From the stress-strain curve, the nanocomposites’ fracture strength (FS), yield strength (YS), and Young’s modulus (YM) were measured.

#### 2.2.4. Differential Scanning Calorimetry (DSC)

The nanocomposites’ thermal stability was characterized using TA instruments (Model Q 200, TA Instruments, New Castle, DE, USA), which was maintained in a controlled environment of N_2_ gas. To avoid oxidation of the samples, 5 mg of the samples were sealed using aluminum pans. These pans were exposed to the heating and cooling cycles. The heating rate was maintained at 10 °C/min. From the generated DSC curves, melting and crystallization points were obtained.

#### 2.2.5. XRD (X-ray Diffraction Spectroscopy)

The developed HDPE nanocomposites were exposed to an XRD diffraction spectrometer (X’Pert3, PANalytical X-ray diffraction system, PANalytical, Denver, CO, USA). The Anode of the instrument was made of copper with CuKα radiation maintained at λ = 1.54 A°. The equipment was run at a voltage of 45 kV and a current of 40 mA. The interplanar distance between the molecules is given by Braggs law [53].

*n*λ = 2*dsin*θ (1)

To evaluate the crystalline size of the nanocomposites, the Scherrer equation is used [54].

(2) $$L_{hkl}=\frac{K\lambda }{\beta \cos\theta \;}$$

where K is the shape factor for the crystal thickness and is taken to be 0.9, β is the half-height of the diffraction peak (rad), λ (nm) is the wavelength, and θ is Bragg’s angle.

The degree of crystallinity is calculated as follows [55]:

(3) $$X_{C}=\frac{I_{110}+1.46{\;I}_{200}}{I_{110}+1.46I_{200}+0.75I_{a}}*100\%$$

where I_110_ and I_200_ are the integral intensity of (110) and (200) lattice planes for respective diffraction peaks. X_c._ is the degree of crystallinity, and I_a_ is the integral intensity of the amorphous phase.

### 2.3. Screening of the Factors

#### 2.3.1. Procedure and Design of Experiments

Initially, five factors and their interactions (i.e., n-TiO_2_, n-CNCs, M-n-TiO_2_, PEgMAH and the particle size of n-TiO_2_ (21 nm, 150 nm)) were designed by full factorial design with three replicates for improving the responses (i.e., fracture strength, yield strength, Young’s modulus of the HDPE nanocomposites). Significant factors were determined by subjecting these factors and responses to perform the analysis of variance (ANOVA) in the MINITAB 17 statistical software program.

#### 2.3.2. Statistical Design of Experiments

The full factorial design allows for a systematic evaluation of the effect of multiple parameters’ effects and their interaction on the response variables [56,57]. Therefore, a full factorial design was performed to obtain a clear picture of the factors’ influence and their interactions on the developed nanocomposites’ mechanical durability. The factors and the levels chosen in this study are tabulated in Supplementary Table S1. All analyses were performed in triplicate to investigate the experimental error(s).

### 2.4. Modelling of the Factors Using Statistical and Machine Learning Algorithm

**Figure 1:**
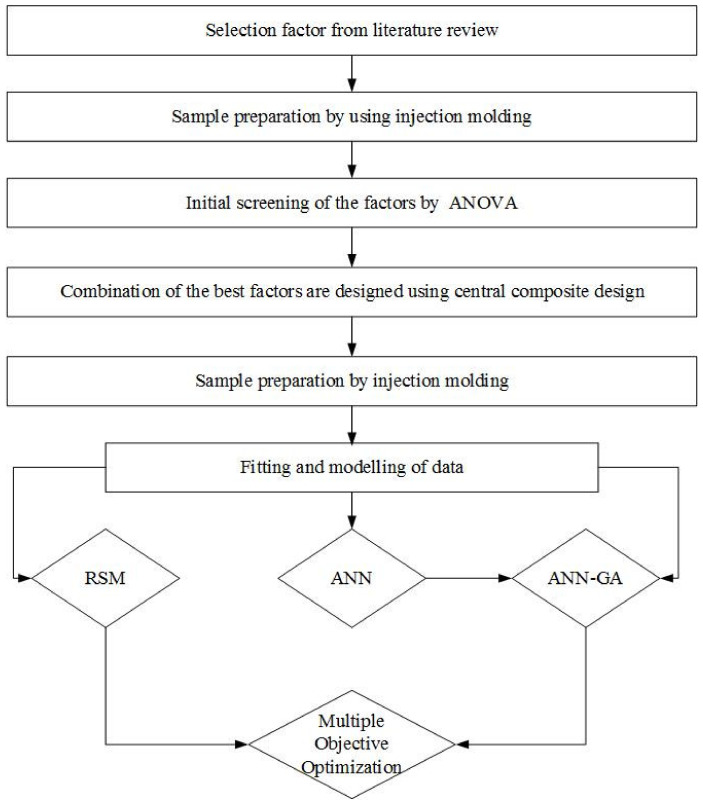
Flow diagram indicating the order of experimental design and the modeling techniques.

The statistical analysis concluded that n-CNCs and n-TiO_2_ were the major contributors to improve the nanocomposites’ fracture strength, yield strength, and Young’s modulus. Their interaction also had a *p* value less than 0.050, which indicates that the factor is significant. Therefore, the experimental design was obtained using a randomized central composite design with two factors (concentration of n-CNCs and n-TiO_2_), three center points, and three replicates. Factors were modeled using the response surface methodology (RSM), artificial neural network (ANN), and genetic algorithms. A sequence of these analyses is illustrated in Figure 1.

#### 2.4.1. Response Surface Methodology (RSM)

**Figure 2:**
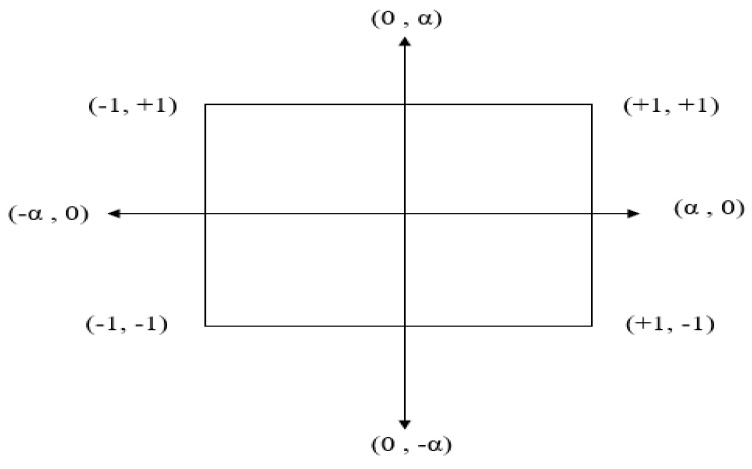
Central composite design for two factors.

Box and Winson [38] developed the central composite design to produce a second-order polynomial equation. This equation facilitates the development of the response surface second-order model and optimizes the influence of n-TiO_2_ nanofiller and n-CNC concentrations on the durability of the developed HDPE-nanocomposites. To design the matrix, a central composite design with two factors, three center points, and three replicated were employed. The number of experiments is determined by 2f + 2f + 1 wherein f is the number of factors. In the experimental design (Figure 2), +1 and –1 indicate the highest value and the lowest value, respectively, and the axial point (α) was kept as the default value, which is 1.414.

Using Minitab 17.0, a second-order polynomial relationship between factors and responses was developed. The second-order polynomial equation is given by

(4) $$Y=\beta_{0}+\overset{k}{\underset{i=1}{\sum }}\beta_{i}X_{i}+\overset{k}{\underset{i,j}{\sum }}\beta_{\mathrm{ij}}X_{i}X_{j}+\overset{k}{\underset{i=1}{\sum }}\beta_{\mathrm{ii}}X_{i}^{2}$$

where β_0_ is the intercept, Y is the response variable, X_i_ (1, 2, 3, …, k) represent the independent variables, β_i_ is the coefficient of the ith factor, β_ij_ represents the coefficient of interaction factor, and β_ii_ is the quadratic factor.

#### 2.4.2. Artificial Neural Network (ANN)

**Figure 3:**
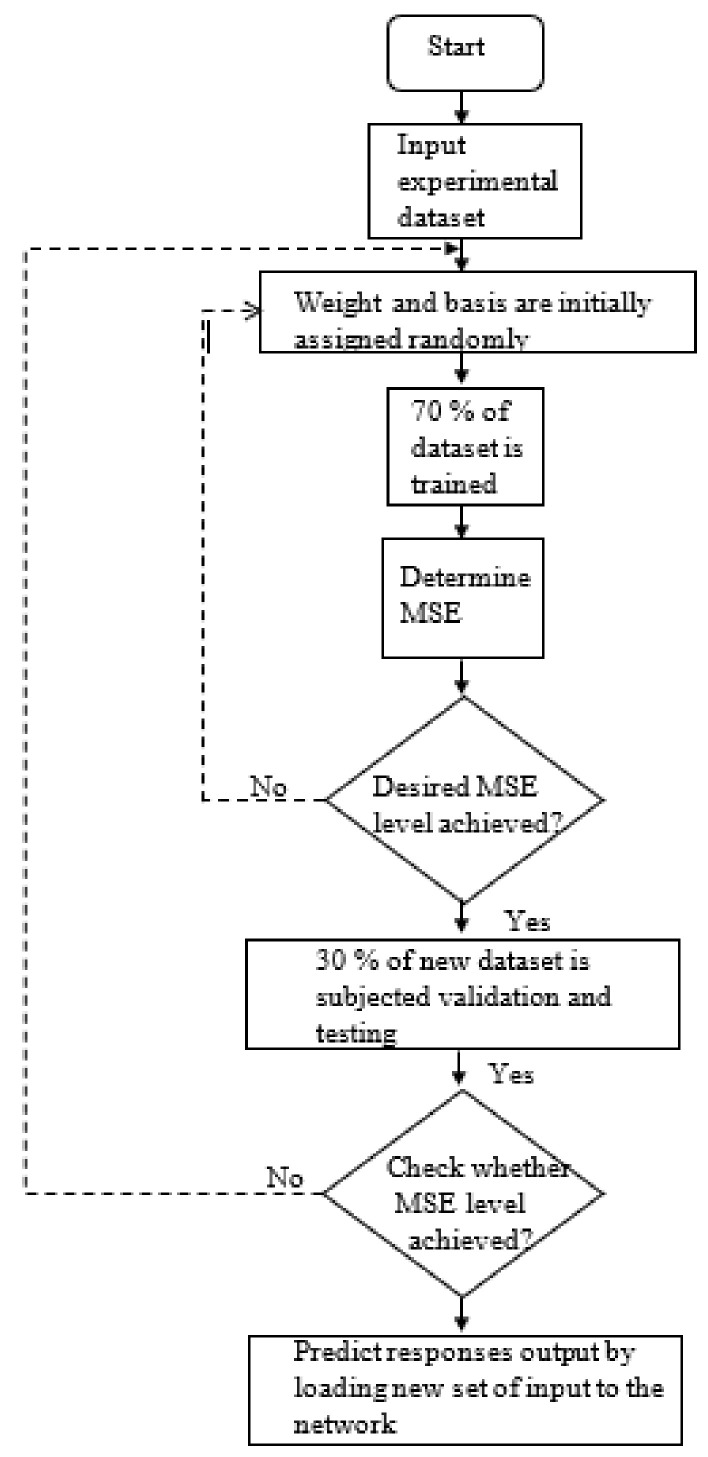
Flow diagram of the artificial neural network used in this study.

**Figure 4:**
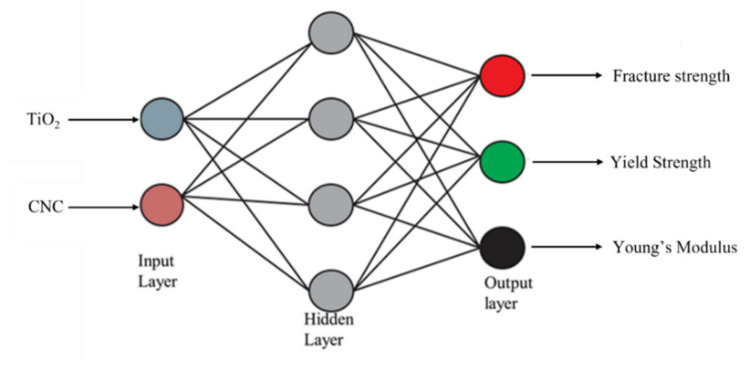
The neural network architecture used in this study.

ANN comprises of several neurons that can be categorized as processing units, classified as input, output, and hidden layers. In this study, data moves only in one direction. Thus, a feedforward network was implemented for this purpose. Information flows from the input layer to the hidden layer and finally to the output layer. The network’s algorithm and architecture play a crucial role in its learning rate and capacity. Among several different algorithms and activation functions of ANN, backpropagation and tansig function are deemed to be more appropriate to process one-directional data of our kind. Since this study’s dataset consists of both input data and target data, a supervised learning type has been chosen. The learning data were distributed randomly in three units training (70%), validation (15%), and testing (15%). In this network, the input layer was the concentration of n-TiO_2_ and n-CNC, while the output layer was the responses such as fracture strength, yield strength, and young’s modulus. The hidden layer and the hidden neuron were fixed by the trial-and-error method. The neural network was designed and processed by Matlab toolbox for the neural network. The steps involved in ANN are schematically represented in Figure 3. The working parameters and the architecture of the neural network used in this study are shown in Supplementary Table S2 and Figure 4. The values of R^2^ and MSE (mean square error) are based on the relations below:

(5) $$R^{2}=\frac{\sum_{i=1}^{N}{\left(\sigma_{i}^{\mathrm{Exp}}-\sigma^{\mathrm{avg}}\right)}^{2}-\sum_{i=1}^{N}{\left(\sigma_{i}^{\mathrm{Exp}}-\sigma_{i}^{\mathrm{ANN}}\;\right)}^{2}}{\sum_{i=1}^{N}{\left(\sigma_{i}^{\mathrm{Exp}}-\sigma^{\mathrm{avg}}\right)}^{2}}$$

(6) $$\mathrm{MSE}=\frac{1}{N}\overset{N}{\underset{i=1}{\sum }}{\left(\sigma_{i}^{\mathrm{Exp}}-\sigma_{i}^{\mathrm{ANN}}\right)}^{2}$$

#### 2.4.3. ANN-Genetic Hybrid Algorithm (ANN-GA)

**Figure 5:**
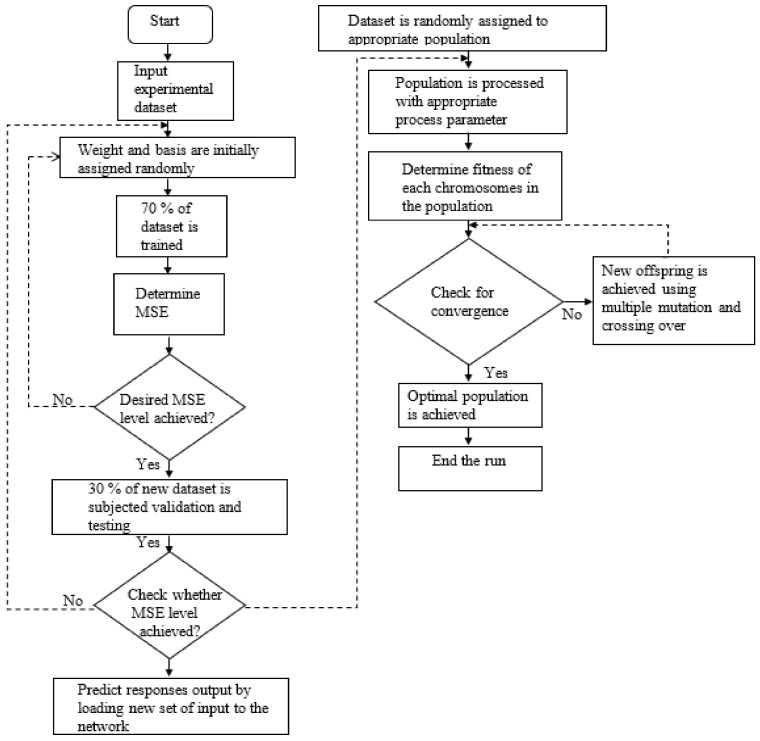
Flow diagram of the artificial neural network-genetic algorithm used in this study.

To carry out the ANN-GA method, neural designer software (Neural Designer, Salamanca, Spain) was implemented. The input of data with the appropriate format was loaded into the software. The input data were differentiated into continuous and categorical variables, and the responses were assigned as the target variables. Target variables can also be subdivided as the used and unused data. The hidden layer, the hidden neuron, and the scaling approach need to be selected to design the neural network. Once the network is recorded, the learning function and its parameters, and the error of interest can be modified. This step is continued several times until a network with the minimum error was obtained. After retrieving the network with minimum error, the network was validated to obtain the network’s global optimization. All steps are presented in Figure 5 as a flow diagram. The data were further processed with two more optimization techniques, such as simulated annealing and the genetic algorithm. The parameters used for these two optimizations are presented in Supplementary Table S3.

## 3. Results and Discussion

Although the combination of multiple nanofillers with polymer matrix has extensive mechanical integrity advantages, this synergy cannot reach its utmost potential due to their deficiency in compatibility [58,59]. The blending of the multiple nanofillers is restricted by the high surface energy of the nanoparticles, leading to the agglomeration of the nanocomposites [9]. On the other hand, the primary purpose of using compatibilizing agents is to enhance the interfacial properties and to improve the nanocomposites’ morphology [60,61]. With these agents, the polymer end region binds to the matrix as they have a similar backbone structure. The maleic anhydride part of the PEgMAH is attracted to the metal oxides’ polar surface [61,62]. The binding of the organosilanes side of MPS to the nanoparticle minimizes the surface energy of the metal oxides, thereby reducing the possibility of agglomeration [63].

The HDPE nanocomposites incorporated with the nanofillers (i.e., TiO_2_, CNC) and compatibilizing agents (i.e., PEgMAH, MPS) were developed using an injection molding machine. In designing nanocomposites for biomedical applications, the materials should acquire excellent durability to accommodate the cells and to provide the structure for their growth and proliferation. In tissue engineering, fracture strength (FS), yield strength (YS), and Young’s modulus (YM) are considered among the best characteristics of the mechanical integrity (i.e., durability) of the materials to be used as biomaterials [7,11]. Hence, in this study, the influence of the nanofillers and compatibilizing agents on the durability of the nanobiocomposites was analyzed using ANOVA.

### 3.1. Screening of the Factors by Analysis of Variance (ANOVA)

The initial screening of the factors (n-TiO_2_, n-CNC, PEgMAH, M-(n-TiO_2_), size of the nanofillers) and the responses (FS, YS, YM) was performed by using analysis of variance (ANOVA). The factors and levels used in designing a factorial design are summarized in Supplementary Table S1. The *p* and F values are estimated by using the degree of freedom, the sum of the square, and the mean square. The probability of the factor and its interaction less than or equal to 0.050 (*p* ≤ 0.050) was considered to be significant. On careful examination of the influence of PEgMAH on the mechanical properties, its role was found to be insignificant, as shown in Supplementary Tables S4–S6. In all instances, the *p*-value of this factor was found to be greater than 0.050 (*p* > 0.050). Moreover, the interaction’s significance was rejected as the factor on its own did not have any significant role in improving the durability of the nanocomposites. However, according to Supplementary Tables S4–S6, the factor M-(n-TiO_2_) and its interaction had a significant role in enhancing the fracture strength, yield strength, and Young’s modulus as the *p*-value came out to be less than 0.050. Further, the factor n-TiO_2_ was found to be significant in improving the responses (i.e., mechanical properties of the nanocomposites), whereas the particle size of the TiO_2_ nanoparticles (i.e., the categorical variable) was not found to be significant, as evident from Supplementary Tables S4–S6 and Figure S3. The responses found with two different sizes of nanoparticles (21 nm and 150 nm) were not much different. Moreover, while comparing the performance of pure n-TiO_2_ and M-(n-TiO_2_) concentrations on improving the nanocomposites’ durability, pure TiO_2_ was found to be better than M-(n-TiO_2_) based on its percentage contribution in fitting the model. On further evaluation of the ANOVA results shown in Supplementary Tables S4–S6, it can be concluded that the CNC concentration has a significant role (*p* < 0.05) in improving the developed nanocomposites’ durability. Moreover, the highest F values for CNC (presented in Supplementary Tables S4–S6) correspond to the fact that it contributed the most to fit the model. This significant improvement of the mechanical strength using CNCs can be attributed to the fact that CNCs on their own have mechanical strength and Young’s modulus close to 167.5 GPa [17].

### 3.2. Thermomechanical Properties of the Nanocomposites

#### 3.2.1. Mechanical Properties of the HDPE Nanocomposites

Further, to develop a second-order model the experiments were run using the central composite design as the design pattern using both TiO_2_ and CNCs as the factors. HDPE nanocomposites samples were developed using an injection molding machine in replicates of three. To deduce the durability of the HDPE nanocomposites, tensile testing was carried out. The fracture strength, yield strength, and Young’s modulus were extracted from the stress-strain curves and summarized in Table 2. The developed nanocomposites’ fracture strength varied from 22.6 to 22.7 MPa, the yield strength was within the range 21.7–27.7 MPa, and the Young’s Modulus was between 314.7 and 815.1 MPa. 

#### 3.2.2. Thermal Behavior of HDPE Nanobiocomposites

##### X-ray Diffraction Spectroscopy XRD

**Figure 6:**
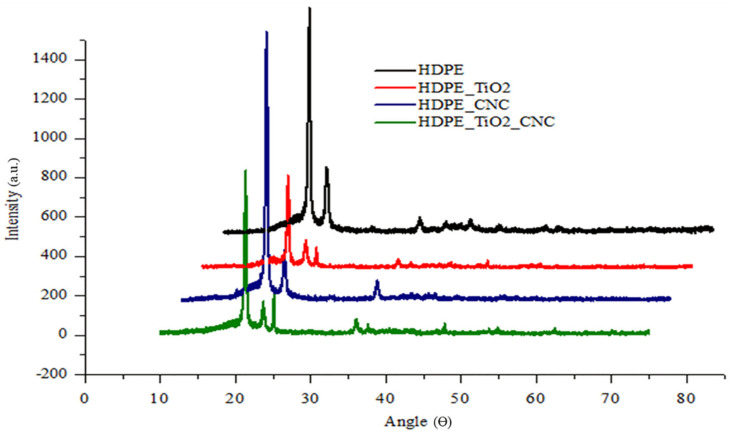
XRD diffractograms of the pure HDPE and HDPE nanobiocomposites.

The effect of titanium dioxide and cellulose nanocrystals was investigated by exposing the HDPE nanocomposites samples to X-ray diffractometry. Prominent X-ray diffraction peaks were visualized at 21.4° and 23.7° corresponding to the peaks found for standard high-density polyethylene [64]. The diffraction peaks indicating the presence of TiO_2_ were located at 25.4°, 37.9°, and 48.1°, which are in correlation with standard peaks of titanium dioxide [18,19]. There is a chance of overlap between the primary diffraction peaks of CNC (2Ɵ = 22.5° and 23°) and HDPE (2Ɵ = 21.4° and 23.7°), as the peaks are at a similar angle. Hence, the CNC peaks cannot be visualized in the graph, but their effect can be interpreted by viewing variation in the crystal size and degree of crystallinity [65]. As shown in Table 3 and Figure 6, the peaks of HDPE with nanofillers have developed more defined peaks. This modification in peaks can be attributed to their crystal size reduction and degree of crystallinity increment. Thereby, it can be concluded that both TiO_2_ and CNC will act as nucleating agents, which can be observed in the form of peaks [18,66]

##### Differential Scanning Colorimetric DSC

**Figure 7:**
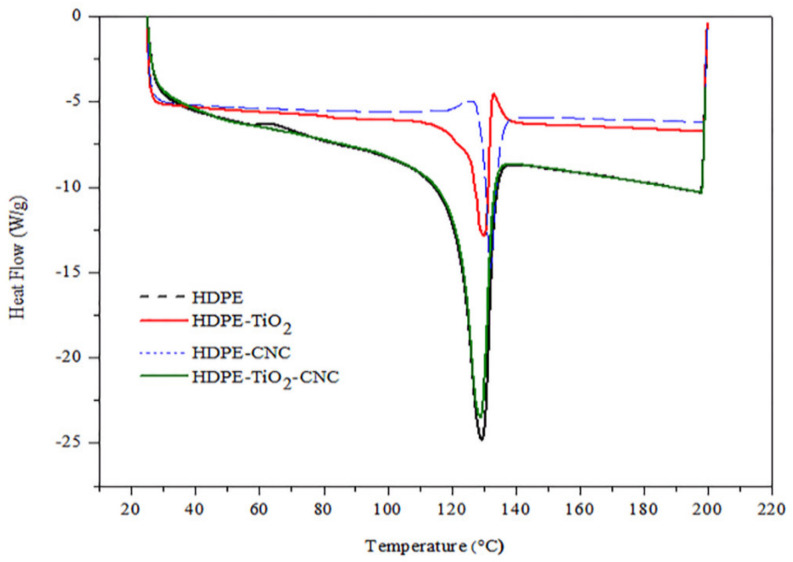
Effect of nanofillers (TiO_2_, CNC, and both) on the melting temperature of the injection-molded HDPE/n-TiO_2_/n-CNC nanobiocomposites.

**Figure 8:**
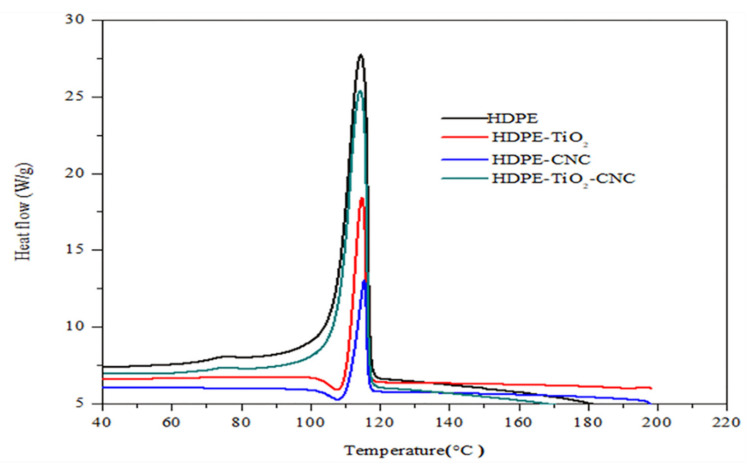
Effect of nanofillers (TiO_2_, CNC, and both) on the crystallization temperature of the injection-molded HDPE/n-TiO_2_/CNC nanobiocomposites.

The melting and crystallization temperatures were obtained upon exposing the pure HDPE and the HDPE nanocomposites to heating (from 25 °C to 200 °C) and cooling cycles (from 200 °C to 25 °C), respectively. The first heating curve was rejected to reduce the probability of the influence of moisture or impurity in the obtained results. As shown in Figure 7 and Figure 8 and Table 4, the melting temperature decreases with the addition of TiO_2_, whereas the crystallization temperature increases compared to the pure HDPE. However, with adding of CNCs to the polymer mix, both the melting and crystallization temperatures increase with respect to that of pure HDPE. The rise in both temperatures may indicate perfect crystallization of the developed nanocomposites [67]. Similarly, Sapkota et al. [68] observed improved thermal stability due to the introduction of both microcrystalline cellulose and cellulose nanocrystals into the low-density polyethylene. However, upon the addition of two nanofillers (5% TiO_2_ and 5% CNCs), a decrease in the crystallization process occurs, as presented in Table 4, which could be attributed to the fact that two nanofillers may have hindered the packing of the molecule chain in polyethylene [66].

According to Table 4, the heat of fusion of the developed HDPE nanobiocomposites decreases when only one nanofiller (either TiO_2_ or CNC) was used. However, when 5 wt.% of each of the nanofillers (TiO_2_ or CNC) were incorporated in the nanobiocomposites, the heat of fusion was greatly elevated. The decrease of the heat of fusion can be attributed to the fact that the addition of a single nanofiller into the polymer matrix hinders the integrity of the crystalline area of the polymeric matrix. However, as the quantity of the nanofiller increases when both nanofillers were added to the matrix, more lamellar crystals are formed by a heterogeneous nucleation process, which then leads to a rise in the crystallization process [1,2,3].

### 3.3. Modelling with Response Surface Methodology (RSM)

From the preliminary screening performed by ANOVA, it was found that CNCs and TiO_2_ nanofillers were the major players in improving the mechanical durability of the developed HDPE nanocomposites. To reduce the probability of data redundancy, randomized experimental runs using these two factors (i.e., CNCs and TiO_2_) were designed using a central composite design. In this design, the range of CNCs and TiO_2_ nanoparticles’ concentration was taken as 0–5.8 wt.% with three center points and three replicates. Data were processed and modeled into a second-order polynomial equation to develop a linear relationship between independent and dependent variables. Table 5 summarizes the model equations and their regression for fracture strength, yield strength, and Young’s modulus of the developed nanocomposites using RSM in Minitab.

**Figure 9:**
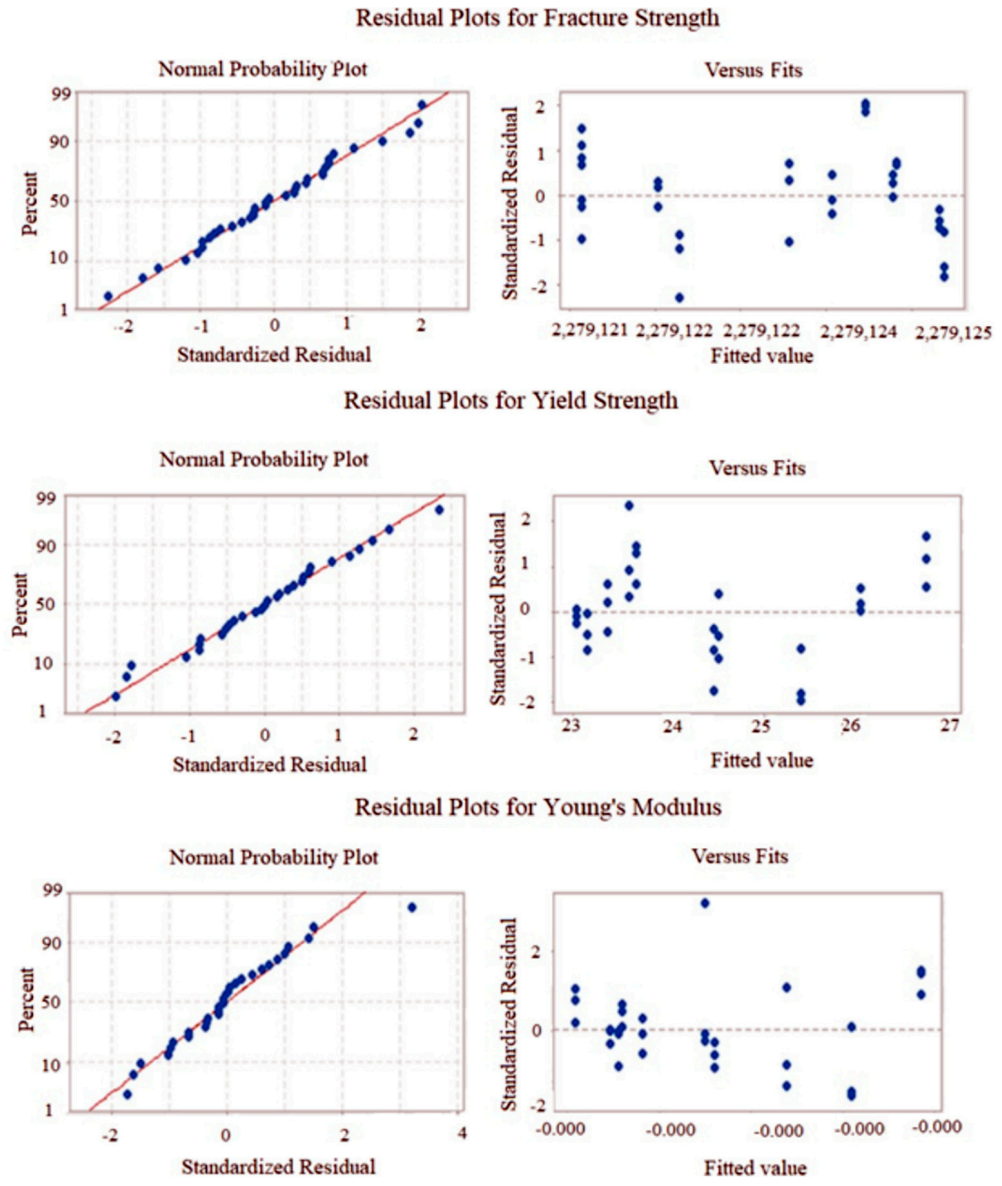
Normality and residual plots obtained from RSM modeled data.

To confirm the models’ adequacy, the regression coefficient, normality plot, and the significance of the model from ANOVA play a significant role. Regression coefficients (R^2^) for fracture strength, yield strength, and Young’s modulus were found to be 73.01%, 92.04%, and 56.64%, respectively. Since the values of the Adjusted R^2^ are not much different than those of R^2^ (Table 5) it can be concluded that improvement of R^2^ has happened because of the goodness of the fit, not just by chance. Even though some of the regression coefficients found in RSM were not high enough, for all of the responses (i.e., FS, YS, YM) the model *p* values were found to be less than 0.05, indicating that the models were significant. Moreover, since most points are passing through the straight line shown in the normality plots presented in Figure 9, the models do not have the normality issue. Figure 9 also contains the residual plots for all three responses used in this study. It is found that the residual plot for any response did not produce any particular shape (i.e., funnel), which in turn confirms that the residual parameter has been satisfied and the models are adequate. On confirmation of the fitted model, using the regression equation, the responses are predicted and summarized in Table 6.

**Figure 10:**
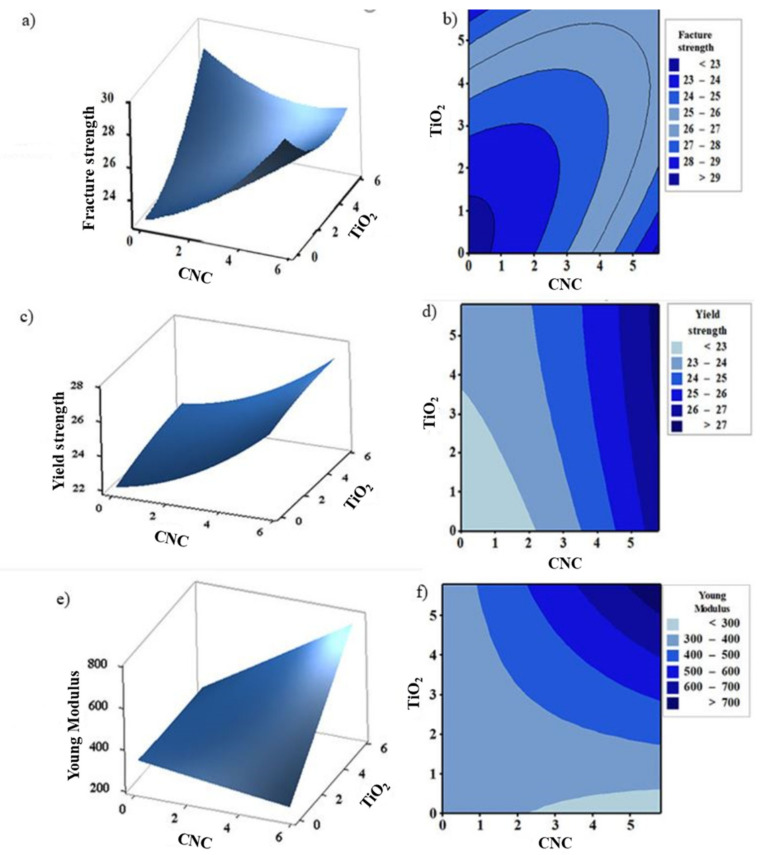
Surface and Contour plots for fracture strength (**a**,**b**), yield strength (**c**,**d**), and Young’s modulus (**e**,**f**) of the developed HDPE nanobiocomposites modeled by response surface methodology (RSM).

The 3D plots (Figure 10) have been produced to visualize the impact of the factors (concentration of CNC and TiO_2_ nanoparticles) on the responses (FS, YS, and YM). In these 3D plots, the interaction between variables has been indicated using the curvatures. Depending on the application of interest, the maximum and minimum values for these responses can be obtained for each factor through the 3D plots [36]. The contour and surface plots for a varying concentration of the factors were plotted for each of the responses. The 3D plot of Figure 10a shows a maximum fracture strength obtained in the presence of higher concentrations of both CNCs and TiO_2_ nanofillers and the minimum fracture strength is obtained when less than 2 wt.% of both factors incorporated in the nanocomposites. The curvatures in Figure 10b indicate a clear interaction between the independent variables. The change in the yield strength of the developed nanocomposites with varying concentrations of both factors is represented in Figure 10c,d. Both contour and surface plots indicate that, with increasing concentrations of TiO_2_ and CNC, higher values of yield strength can be obtained. Similarly, Young’s modulus (Figure 10e,f) can reach its maximum when the concentrations of the factors are at high levels.

### 3.4. Multi-Objective Optimization with Response Surface Methodology Coupled with Genetic Algorithm (RSM-GA)

**Figure 11:**
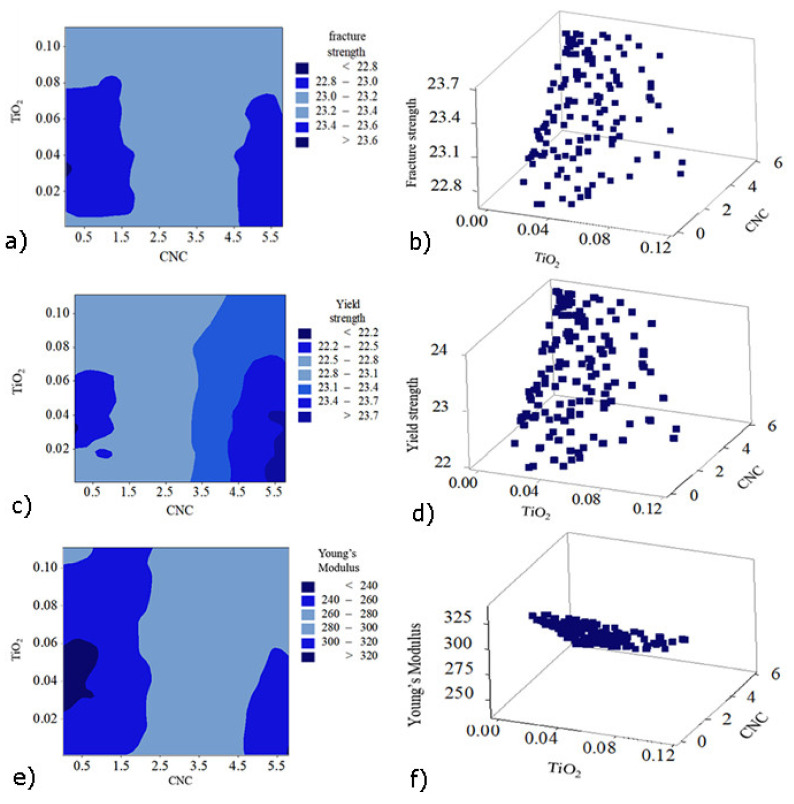
Scatter and Contour plots for simultaneously optimized multi-responses [fracture strength (**a**,**b**), yield strength (**c**,**d**), and Young’s modulus (**e**,**f**)] of the developed HDPE nanobiocomposites modeled by RSM-GA.

It is well known that single response optimization is a tedious and time-consuming approach. The major problem of single response optimization lies in the fact that there is a probability of obtaining the resultant combination of the factors impacting only one of the responses positively while impacting other responses negatively. Multi-objective optimization by implementing both statistical and machine learning approaches can be an excellent method to resolve this concern [69,70]. In this study, a nonlinear and randomized modeling between the three responses (i.e., FS, YS, YM) and two factors (i.e., the concentration of CNCs and TiO_2_ nanofillers) have been carried out using Optimization tools application with the gamultiobj function in MATLAB simulation system. The regression equations, known as fitness equations, obtained from the response surface methodology (shown in Table 5) have been used as the base equations to optimize the responses. A random population was initially assigned using the fitness equations and the with help of lower and upper bounds of the variables. This study’s population type is a double vector, and the population size was set at 150. The population was allowed to crossover at a ratio of 0.8. The crossing over function was allowed at the intermediate stage, and the maximum generations were assigned at 150. The resulting population scores are plotted and represented in a surface plot in Supplementary Figure S4. This shows that the improvement in Young’s modulus and yield strength of the developed nanocomposites has been achieved at the expense of fracture strength of the nanocomposites. Figure 11a–f represent the scatter and contour plots of the factors with each response (fracture strength (a,b), yield strength (c,d), and Young’s modulus (e,f)) modeled by the RSM-GA. As observed in Figure 11a,b, the incorporation of 1.0 wt.% of n-TiO_2_ and 5.74 wt.% CNCs to the polymer matrix produced the maximum yield strength of 23.9 Mpa, whereas the highest value of fracture strength was obtained as 23.6 MPa by combining HDPE with 0.03 wt.% n-TiO_2_ and 5.74 wt.% of CNCs (shown in Figure 11c,d). The maximum Young’s modulus achieved was 327 MPa when the concentration of TiO_2_ and CNCs were used at 0.04 wt.% and 0.60 wt.%, respectively (Figure 11e,f). The R^2^ for the regression equations for FS, YS, and YM were found to be 68.52%, 91.14%, and 49.17%, respectively. Although R^2^ is recommended to be higher than 95% to achieve better-predicted data, our data could not achieve such a value. This could explain the difference between the experimental and predicted results [71,72].

### 3.5. Modelling with Artificial Neural Network (ANN)

**Figure 12:**
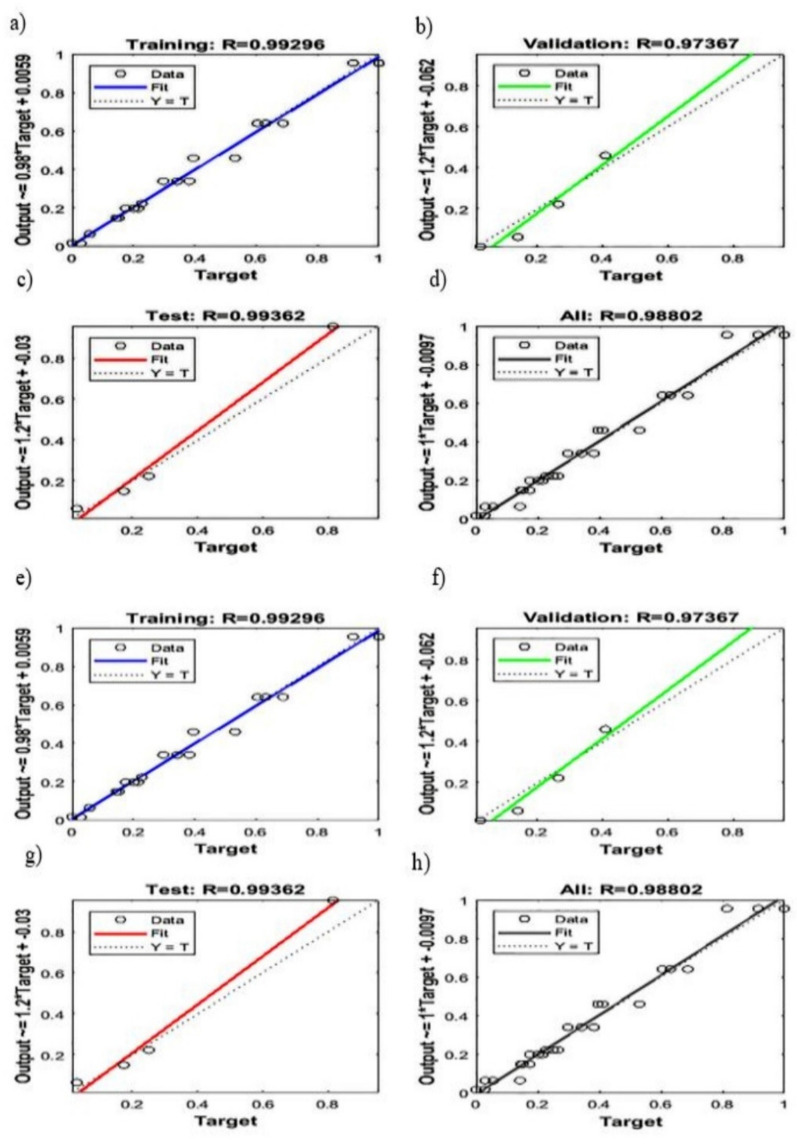
Regression plots for the fracture strength modeled by ANN ((**a**): training, (**b**): validation, (**c**): testing, (**d**): average) and the yield strength of the developed nanobiocomposites modeled by ANN ((**e**): training, (**f**): validation, (**g**): testing, (**h**): average).

**Figure 13:**
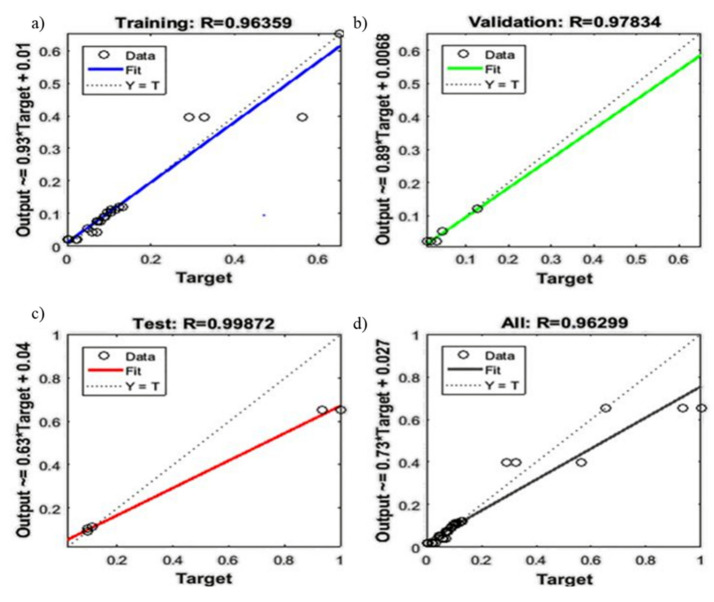
Regression plots for Young’s modulus modeled of the developed nanobiocomposites by ANN ((**a**): training, (**b**): validation, (**c**): testing, (**d**): average).

The artificial neural network (ANN) is a special type of machine learning algorithm that is modeled after the human brain. ANN is typically used to develop a nonlinear relationship between the factors and the responses. Initially, the parameters such as weights and biases are assigned at random. The pattern between the factors and responses is determined, and a new sample set is loaded to the network to obtain the predicted target values. Then, the obtained predicted target values are checked with the actual target values, and the difference (i.e., error) is noted. Depending on the error, the weight and biases are being updated. The network is being run several times until the R^2^ is close to 1. The mean square error for that network is at its minimum [73,74]. In this study, the neural network was developed using NN ToolBox in MATLAB using a feedforward multilayer perceptron approach. After running several different algorithms, the Levenberg-Marquardt algorithm was found to be the most suitable one for the data used in the study. as this algorithm exhibited minimum error. Both the input and output variables were initially stored in MATLAB and using the neural network toolbox, a network with a varying number of hidden neurons and hidden layers was developed. Initially, the network was run, keeping the weight and biases at random. Using the trial-and-error method, the most efficient hidden layer and hidden neurons were obtained. Depending on the R^2^ and the mean square error, the network was being saved or rejected. The process parameters such as Epochs, Mu, and gradients were being adjusted to obtain the network with the least mean square error. Once the most suitable network was retrieved, the network was rerun with a new set of input given to the network. The network’s prediction of the output was stored and compared with the experimental data. The R^2^ for fracture strength, yield strength, and Young’s modulus of the HDPE nanocomposites were found to be 0.97491, 0.98802, and 0.96299, respectively, confirming that the designed network is adequate to generate the optimum predicted outputs with minimum errors, as shown in Figure 12 and Figure 13. The outputs of the predicted and experimental results for all of the responses (i.e., YS, FS, YM) are summarized in Table 7. The mean square error for fracture strength, yield strength, and Young’s modulus were found to be 2.5 × 10^−2^, 2.8 × 10^−2^, and 6.9 × 10^−2^, respectively, further confirming the adequacy of the model.

Equation (5) is used to present the relationship between the factors and responses in the neural network [75]. The weights and biases of the designed neural network are summarized in Supplementary Table S7. The output can be predicted by substituting the hidden layers’ weights and biases and the hidden neurons into Equation (7)

(7) $$Y=B1+\mathrm{LW}\times \mathrm{TAN}\mathrm{SIG}\left(B2+\mathrm{IW}\times X\right)$$

where *Y* is the response variable; X is the input variable, LW is the weights corresponding to the connection assigned between the hidden layers to the output layer, IW represents the weights corresponding to the connection assigned between the input layers to hidden layers, while B1 and B2 denote bias vectors for the output and hidden layers.

#### Validation of Artificial Neural Network Data by Genetic Algorithm

**Figure 14:**
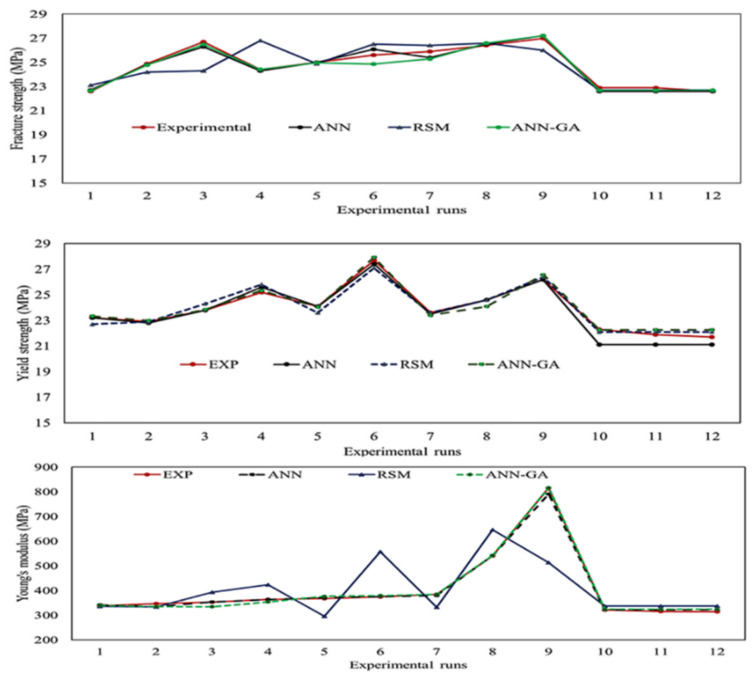
Comparison of the outputs of the modeling techniques with the experimental results.

The artificial neural network is known to be a reliable technique when fitting nonlinear and ill-fitted data. However, local optimization and overfitting are the two major concerns of artificial neural network-based optimization. The hybrid of artificial neural networks and genetic algorithms was implemented to achieve global optimization and to reduce data overfitting [76]. In this approach, initially, the data are assigned randomly to a particular population of neural networks. Upon running the simulation, each population tries to optimize the target for the input database. These data are then stored in the form of a chromosome, in which the locus of the chromosome represents one subunit of the neural network. Using chromosomes as a reference, a new set of the population is assigned. The new population is decided based on the concept of the ‘survival of the fittest’ while the chromosome or network that provides the highest fitting value is considered for the next run. The current population undergoes crossover and mutation to design an improved neural network [77]. These steps are being continued until a network with minimum error is obtained. Finally, a fresh set of test samples is added to this network to check the designed network’s efficiency. The predicted output ANN-GA is stored and compared with the predicted output of ANN. Figure 14 shows almost no difference in the predicted results obtained from both ANN and ANN-GA, which indicates that the designed neural network did not show any overfitting and the optimized results were not locally optimized.

### 3.6. Comparison between RSM and ANN Model

**Figure 15:**
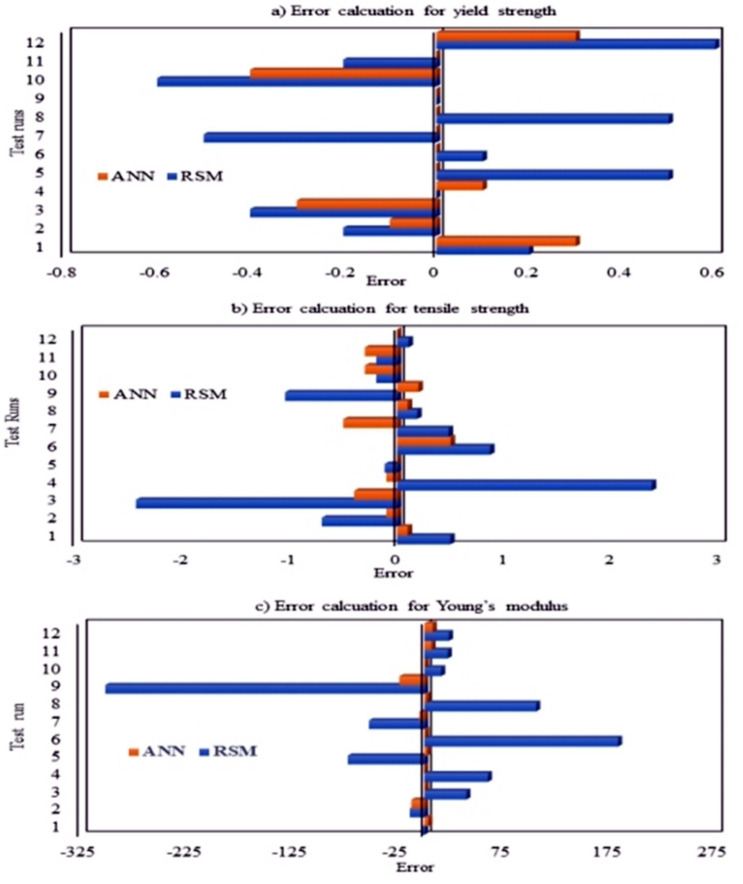
Bar charts representing the predicted errors of (**a**) yield strength; (**b**) fracture strength and (**c**) Young’s modulus of the developed nanocomposites by ANN and RSM.

Both RSM (statistical method) and ANN (machine learning algorithm) are powerful techniques to develop a relationship between the dependent and independent variables. Both of these models were able to predict the results by fitting the experimental data. The difference between experimental and predicted values (i.e., errors) is used to check model efficacy. As shown in Figure 15, errors were higher in the case of the RSM predicted results than that of ANN. Comparing the coefficient of regression for all three responses (YS, FS, YM) modeled with RSM (Figure 10 and Figure 11, Supplementary Figure S4, and Table 6) and ANN (Figure 12 and Figure 13 and Table 7), it is evident that ANN is a better technique than RSM to model the set of data used in this study to determine the influence of nanofiller concentration on the properties of HDPE nanocomposites. The R^2^ values obtained by RSM were 73.01% (FS), 92.04% (YS), and 56.64% (YM), but the R^2^ values were above 98% for all three responses modeled by ANN, as shown in Figure 12 and Figure 13. Similar results were obtained regarding the impact of clay and EPDM (ethylene–propylene diene monomer) for optimizing the mechanical properties of polypropylene using the RSM and ANN methods. By modeling and predicting the data using RSM and ANN, Nakhaei et al. [78] concluded that the ANN provided an adequate interpretation of the responses. Moghri et al. [69,70] predicted the fracture modulus for composites prepared using polyamide-6/nano clay using RSM and ANN methods and reported that ANN was adequate to model the data with a sample size of 20% less. On the contrary, similar effectiveness of both RSM and ANN has been reported in predicting the responses [29,79] while others have found that the RSM is better than ANN in modeling [80].

### 3.7. Multi-Objective Optimization Using Artificial Neural Network-Genetic Algorithm (ANN-GA)

**Figure 16:**
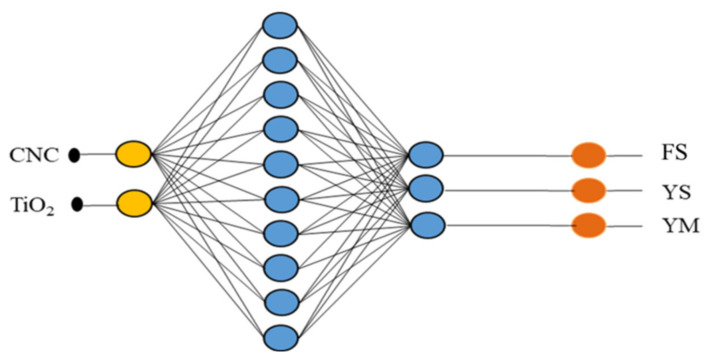
Final Architecture of ANN- GA network.

**Figure 17:**
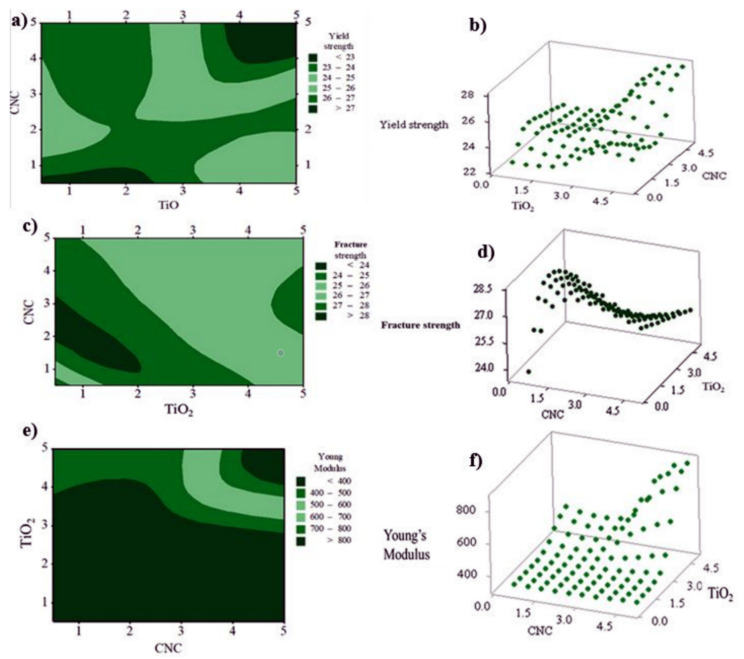
Scatter and contour plots for multi-optimized responses (fracture strength (**a**,**b**), yield strength (**c**,**d**), and young’s modulus (**e**,**f**)) of the developed HDPE nanobiocomposites modeled by ANN-GA Hybrid.

The above analysis confirmed that the ANN-GA machine learning optimization technique is found to be the most reliable technique to model the type of data used in this study. Although ANN is the recommended tool to optimize the nanofiller concentrations for the mechanical durability of the HDPE nanocomposites, there is a high probability of overfitting and local optimization. To confirm the fitting and to attain global optimization, the genetic algorithm plays an important role. In this study, multi-objective optimization was performed using a combination of artificial neural networks and genetic algorithms. Initially, a network was developed between the two factors and three responses, and after conducting the trial-and-error method, the number of hidden neurons and hidden layers were obtained. Upon training the developed network and obtaining the minimum mean square error, the data was computed to a genetic algorithm network. Parameters employed for the genetic algorithm were obtained by trial-and-error approach. Once the best network was obtained, the fresh input was loaded into the software as shown in the genetic algorithm architecture in Figure 16. The predicted outputs generated by the ANN-GA hybrid technique are illustrated in Figure 17a–f. Using the ANN-GA method, the highest fracture strength (28 MPa) was obtained with the addition of 5.0 wt.% of CNCs along with 4.0 wt.% of TiO_2_ to the HDPE polymer matrix (Figure 17a,b). The maximum yield strength obtained from the network was 27.9 MPa when the concentration of TiO_2_ was about 4 wt.% and CNCs 5 wt.% (Figure 17c,d), while upon the addition of 5 wt.% of each of TiO_2_ and CNCs nanofiller to the polymer matrix, it was predicted that Young’s modulus would be 870 MPa (Figure 17e,f). Upon running the multiple objective optimizations by ANN-GA and RSM, the differences in error(s) are represented using the bar plots in Figure 15. It is evident that there was only a slight variation between the experimental run and predicted results found in ANN-GA, but the differences in RSM are significant.

## 4. Conclusions

While developing nanobiocomposites, thermomechanical characteristics play a crucial role in their performance depending on the application of interest. Polyethylene has proven to be an excellent candidate for the polymer matrix in bone and cartilage tissue engineering. However, achieving mechanical integrity similar to the natural tissue or organ is still an unresolved issue. A wide range of ingredients (i.e., nanofillers) is commonly incorporated into the polymer matrix to improve the mechanical durability of the nanocomposites on a trial-and-error basis. Nevertheless, this trial-and-error method is tedious, expensive, and at times unreliable. However, the statistical and machine learning techniques can be conveniently used to minimize the time and expenses required for rigorous experimentation. As per our knowledge, systematic multivariable optimization of the key factors that improve the mechanical durability of the HDPE nanobiocomposites using both statistical and machine learning algorithms has not yet been reported. The current study can be concluded as follows:

(1) Initially, five factors (i.e., n-TiO_2_, n-CNC, PEgMAH, M-(n-TiO_2_), size of the nanofillers), and three responses (yield strength, fracture strength, and Young’s modulus) were chosen and screened for the insignificant factors by ANOVA using a full factorial design experiment. The concentrations of TiO_2_ and CNCs nanofillers were found to be the significant factors to improve the mechanical durability of the HDPE nanobiocomposites.

(2) Upon confirming the significant factors, a central composite design was used to develop a new set of experiments using the significant factor (i.e., TiO_2_ and CNCs nanofillers) only. The resulting data were then modeled using the response surface methodology (RSM) and artificial neural network (ANN). Due to its ability to predict the new inputs, ANN has been found to be better than RSM in predicting the mechanical properties of the developed nanobiocomposites by varying the two significant factors (i.e., TiO_2_ and CNCs nanofiller).

(3) Later in this study, the same data were optimized with ANN-GA and it was found that the predicted data obtained from ANN and ANN-GA are more or less the same, confirming that the modeled dataset was not locally optimized and/or overfitting did not occur.

(4) Finally, the ANN-GA multi-objective optimization predicted the mechanical properties of the developed HDPE nanobiocomposites similar to the experimental ones, and the maximum fracture strength, yield strength, and Young’s modulus were predicted to be 28 MPa, 27.9 MPa, and 670 MPa, respectively, upon the incorporation of TiO_2_ and CNC nanofillers of less than 5 wt.%.

Based on the modeled results, the proposed approach may be considered a promising method to optimize the amount and type of nanofillers that need to be added to the polymer matrix. The responses (i.e., mechanical integrity) can be safely predicted through this procedure for any chosen concentration of TiO_2_ and CNC nanofillers.

## Figures and Tables

**Table 1:** Processing conditions of the injection molding technique used for the fabrication of the HDPE nanocomposites.

Variables	Parameter
Barrel temperature	150 (°C)
Mold temperature	100 (°C)
Holding time in barrel	50 (min)
Injection pressure (bar)	3–4 (bar)

**Table 2:** The mean mechanical properties of the developed HDPE nanobiocomposites.

CNC	TiO_2_	Fracture Strength (MPa)	Yield Strength (MPa)	Young’s Modulus (MPa)
1	1	22.6	23.2	347.3
0.17	3	24.9	22.9	338.1
3	3	26.7	23.8	363.9
5	1	24.4	25.2	385.2
3	0.17	25	24.1	367.8
5.8	3	25.6	27.7	815.1
1	5	25.9	23.6	353.7
3	5.8	26.4	24.6	375
5	5	27	26.2	540.5
0	0	22.9	22.3	322
0	0	22.9	21.9	315.8
0	0	22.6	21.7	314.7

**Table 3:** XRD results for the HDPE/TiO_2_/CNC nanobiocomposites.

Sample No.	Sample Name	TiO_2_ (%)	CNC	Crystal Planes	2Ɵ (°)	*L_hkl_*	Degree of Crystallinity (%)
1	HDPE	0	0	110	21.29	23.2	93.7
				200	23.65	17.26	
2	HDPE-TiO_2_	5	0	110	21.33	24.4	95
				200	23.67	15.22	
3	HDPE-CNC	0	5	110	21.29	24.4	96.3
				200	23.65	15.2	
4	HDPE-TiO_2_ -CNC	5	5	110	21.31	24.4	96.9
				200	23.65	13.9	

**Table 4:** DSC results for the HDPE/TiO_2_/CNC nanobiocomposites.

Sr. No.	Sample	Melting Temperature (°C)	Crystallization Temperature (°C)	Heat of Fusion (J/g)
1	HDPE	128.92	114.22	170.0
2	HDPE-n-TiO_2_	127.48	114.86	127.8
3	HDPE-n-CNC	131.96	115.33	45.47
4	HDPE-n-TiO_2_-n-CNC	127.86	114.22	175.0

**Table 5:** The models developed by RSM.

Response	R^2^	Adjusted R^2^	Equations
Fracture strength (MPa)	73.01%	68.52%	22.707 + 0.364 CNC − 0.039 TiO_2_ + 0.1353 CNC*CNC + 0.1860 TiO_2_*TiO_2_ + 0.2564 CNC*TiO_2_
Yield Strength (MPa)	92.40%	91.14%	22.067 + 0.216 CNC + 0.335 TiO_2_ + 0.0946 CNC*CNC − 0.0214 TiO_2_*TiO_2_ +0.0116 CNC*TiO_2_
Young’s Modulus(MPa)	56.64%	49.17%	337.9–17.3 TiO_2_ − 3.5 CNC + 0.21 TiO_2_*TiO_2_ + 0.44 CNC*CNC + 15.83 TiO_2_*CNC

**Table 6:** Summarized predicted mechanical properties of the HDPE nanobiocomposites by using RSM equations.

CNC	T_i_O_2_	Fracture Strength (MPa)	Yield Strength (MPa)	Young’s Modulus (MPa)
1	1	23.1	22.7	333.6
0.17	3	24.2	22.9	336.5
3	3	24.3	24.3	423.9
5	1	26.8	25.8	333
3	0.17	24.9	23.6	295.5
5.8	3	26.5	27.1	513.8
1	5	26.4	23.6	393.4
3	5.8	26.6	24.6	557.9
5	5	26.0	26.4	646.1
0	0	22.7	22.1	337.9
0	0	22.7	22.1	337.9
0	0	22.7	22.1	337.9

**Table 7:** Summarized predicted mechanical properties of the HDPE nanobiocomposites by ANN.

CNC	TiO_2_	Fracture Strength (MPa)	Yield Strength (MPa)	Young’s Modulus (MPa)
1	1	22.7	23.2	340.1
0.17	3	24.8	22.8	341.9
3	3	26.3	23.8	363.5
5	1	24.3	25.6	380.5
3	0.17	25.0	24.1	370.5
5.8	3	26.1	27.4	791.5
1	5	25.4	23.5	354.3
3	5.8	26.5	24.6	375.8
5	5	27.2	26.2	542.7
0	0	22.6	21.1	322.7
0	0	22.6	21.1	322.7
0	0	22.6	21.1	322.7

## Data Availability

The data presented in this study are available within this article.

## Supplementary Materials

The following are available online at https://www.mdpi.com/article/10.3390/polym13183100/s1, Figure S1. Schematic illustration of HDPE-TiO_2_ grafting with maleic anhydride. Figure S2: Schematic representation of the binding mechanism of TiO_2_ to saline coupling agent (MPS). Figure S3. Pie chart showing the influence of the factor (nanoparticles’ size) on: (a) fracture strength (b) yield strength and (c) Young’s modulus. Figure S4. Surface plot comparison between the responses using RSM-GA. Table S1. Factors and levels used for ANOVA analysis; Table S2 Working parameters for ANN model. Table S3: Working parameters for GA-ANN model. Table S4. Summary of ANOVA results for yield strength. Table S5. Summary of ANOVA results for fracture strength. Table S6. Summary of ANOVA results for Young’s modulus. Table S7. Summarized data containing weights and biases of the neural networks.
