# Supplementary material for: Statistical and Machine Learning-Driven Optimization of Mechanical Properties in Designing Durable HDPE Nanobiocomposites
Source: Polymers (Basel). 2021 Sep 15;13(18):3100. doi: 10.3390/polym13183100 (PMC8472960; doi:10.3390/polym13183100)
Supplement: Supplementary file 1 [file polymers-13-03100-s001.zip › polymers-1346544-supplementary.pdf]

Supplementary Figures:

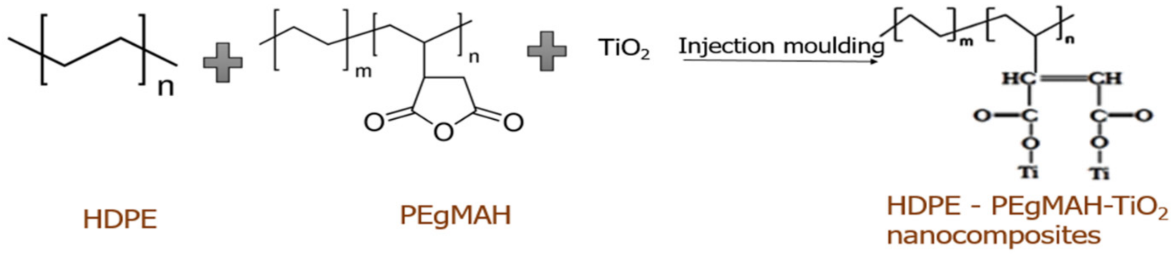

Figure S1. Schematic illustration of HDPE-TiO_2 grafting with maleic anhydride

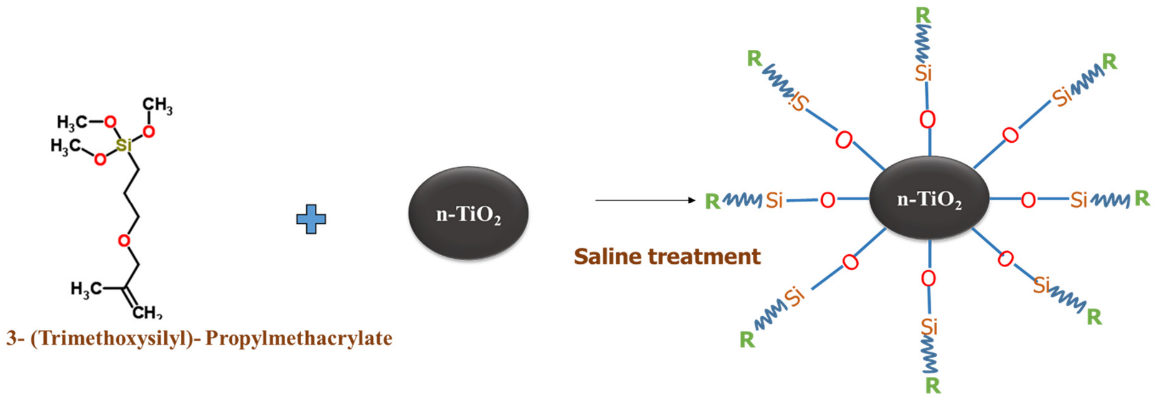

Figure S2: Schematic representation of the binding mechanism of TiO_2 to saline coupling agent (MPS).

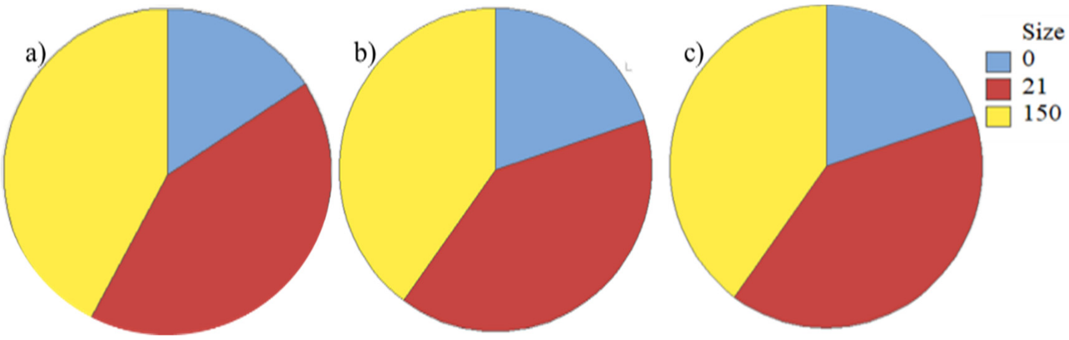

Figure S3. Pie chart showing the influence of the factor (nanoparticles' size) on: a) fracture strength b) yield strength and c) Young's modulus.

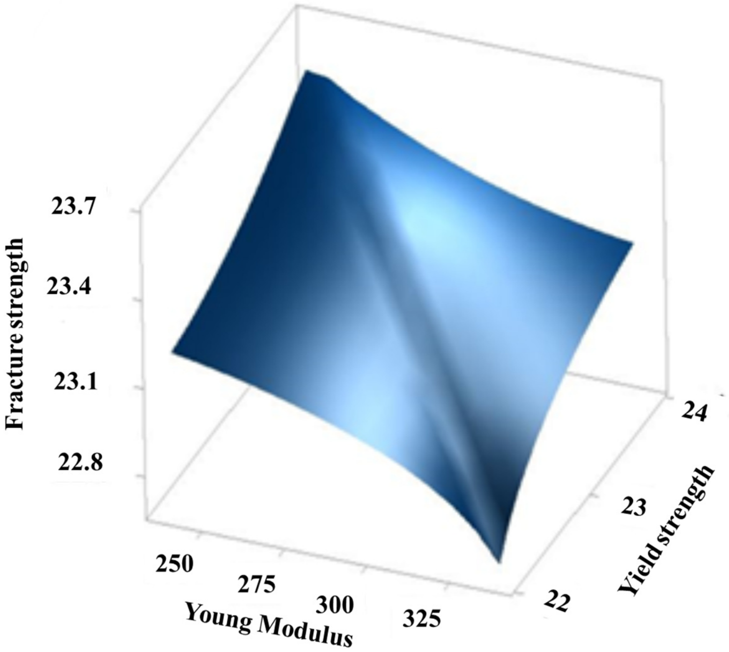

Figure S4. Surface plot comparison between the responses using RSM-GA.

Supplementary Tables:

Table S1. Factors and levels used for ANOVA analysis.

Samples	Factors	Abbreviation	Number of levels	Level 1	Level 2
A	Titanium dioxide nanofillers (%)	n-TiO_2	2	0	5
B	Modified titanium dioxide (%)	M-(TiO_2)	2	0	5
C	Size of the nanoparticles (nm)	Nm	2	21	150
D	Polyethylene grafted maleic anhydride (%)	PEgMAH	2	0	3
E	Cellulose nanocrystals (%)	n-CNC	2	0	5

Table S2. Working parameters for ANN model.

Variables	Parameter
Hidden Layer	1- 20 neurons
Method	Feed forward back propagation
Function	Tansig
Adaption learning function	Levenberg-Marquardt backpropagation
Performance function	Mean square error
Maximum number of epochs	1000-2000
Mu	0.01
Mu (max)	10000000000
Gradient	1e-05-1e-07

Table S3. Working parameters for GA-ANN model.

**Variables**	**Parameter**
Hidden Neurons	1- 20 neurons
Hidden neurons	2
Function	Tansig
Adaption learning function	Levenberg-Marquardt backpropagation
Performance function	Mean square error
Order of selection	Simulated annealing
Maximum number of epochs	1000-2000
Mu	0.01
Populations	150
Learning rate	Medium
Mutation rate	Medium
Trail No	1-10
Maximum generation	150

Table S4. Summary of ANOVA results for yield strength.

Analysis of Variance of Yeild strength

Source	DF	Seq SS	Contribution	Adj SS	Adj MS	F-Value	P-Value
TiO2	1	4.055	1.81%	15.243	15.243	18.77	0.000
m-TiO2	1	12.995	5.79%	12.995	12.995	16.00	0.000
PEgMAH	1	0.174	0.08%	0.845	0.845	1.04	0.313
NCC	1	134.976	60.11%	105.536	105.536	129.95	0.000
TiO2*PEgMAH	1	1.601	0.71%	0.107	0.107	0.13	0.719
TiO2*NCC	1	3.577	1.59%	0.426	0.426	0.52	0.472
m-TiO2*PEgMAH	1	3.627	1.62%	3.627	3.627	4.47	0.040
m-TiO2*NCC	1	1.290	0.57%	1.290	1.290	1.59	0.214
PEgMAH*NCC	1	17.429	7.76%	7.508	7.508	9.24	0.004
TiO2*PEgMAH*NCC	1	0.002	0.00%	2.704	2.704	3.33	0.074
m-TiO2*PEgMAH*NCC	1	5.825	2.59%	5.825	5.825	7.17	0.010
Error	48	38.983	17.36%	38.983	0.812		
Total	59	224.535	100.00%				

Model Summary

S	R-sq	R-sq(adj)	PRESS	R-sq(pred)
0.901196	82.64%	78.66%	62.0051	72.39%

Table S5. Summary of ANOVA results for fracture strength.

Analysis of Variance

Source	DF	Seq SS	Contribution	Adj SS	Adj MS	F-Value	P-Value
Model	11	122.662	83.08%	122.662	11.1511	20.09	0.000
Linear	5	93.872	63.58%	88.096	17.6193	31.74	0.000
TIO2	1	1.204	0.82%	12.163	12.1634	21.91	0.000
M-TiO2	1	1.229	0.83%	6.560	6.5600	11.82	0.001
SIZE	1	11.117	7.53%	9.813	9.8133	17.68	0.000
PEGMAH	1	6.103	4.13%	0.578	0.5779	1.04	0.313
NCC	1	74.219	50.27%	66.247	66.2470	119.36	0.000
2-Way Interactions	6	28.791	19.50%	28.791	4.7984	8.65	0.000
TIO2*SIZE	1	3.497	2.37%	2.115	2.1154	3.81	0.057
TIO2*PEGMAH	1	2.409	1.63%	17.113	17.1131	30.83	0.000
TIO2*NCC	1	4.244	2.87%	6.735	6.7345	12.13	0.001
M-TiO2*PEGMAH	1	15.746	10.67%	18.123	18.1233	32.65	0.000
M-TiO2*NCC	1	0.901	0.61%	1.822	1.8220	3.28	0.077
PEGMAH*NCC	1	1.993	1.35%	1.993	1.9933	3.59	0.065
Error	45	24.977	16.92%	24.977	0.5550		
Lack-of-Fit	6	12.790	8.66%	12.790	2.1317	6.82	0.000
Pure Error	39	12.187	8.25%	12.187	0.3125		
Total	56	147.639	100.00%				

Model Summary

S	R-sq	R-sq(adj)	PRESS	R-sq(pred)
0.745008	83.08%	78.95%	38.7158	73.78%

TableS6. Summary of ANOVA results for Young's modulus.

```
Analysis of Variance of Young's Modulus

Source                DF   Seq SS  Contribution   Adj SS   Adj MS  F-Value  P-Value
  Tio2                 1     2029         0.15%    22611    22611     4.27    0.044
  m-tio2               1    76133         5.49%    76133    76133    14.36    0.000
  PEgMAH               1    19110         1.38%    10385    10385     1.96    0.168
  NCC                  1   698212        50.37%   475922   475922    89.77    0.000
  Tio2*PEgMAH          1     3534         0.25%     3855     3855     0.73    0.398
  Tio2*NCC             1   153575        11.08%    72447    72447    13.67    0.001
  m-tio2*PEgMAH        1      711         0.05%      711      711     0.13    0.716
  m-tio2*NCC           1     1184         0.09%     1184     1184     0.22    0.639
  PEgMAH*NCC           1    25027         1.81%    85148    85148    16.06    0.000
  Tio2*PEgMAH*NCC      1   101163         7.30%   150302   150302    28.35    0.000
  m-tio2*PEgMAH*NCC    1    51044         3.68%    51044    51044     9.63    0.003
Error                 48   254471        18.36%   254471     5301
  Lack-of-Fit          8   154426        11.14%   154426    19303     7.72    0.000
  Pure Error          40   100045         7.22%   100045     2501
Total                 59  1386192       100.00%


Model Summary

      S    R-sq  R-sq(adj)   PRESS  R-sq(pred)
72.8112  81.64%     77.44%  395858      71.44%
```

Table S7. Summarized data containing weights and biases of the neural networks.

Neural network weight and bias for Fracture strength				
IW		LW	B1	B2
1.3208	-4.3184	0.29061	-4.3173	-0.57446
-1.2033	3.9163	0.74794	3.7739	
0.26228	4.4169	0.40458	-2.4685	
-4.2226	-0.59281	-0.15949	1.6306	
-4.4142	0.22458	-0.59125	-0.02313	
-3.7963	-2.3729	0.77764	-0.748	
-2.9371	3.2325	0.22993	-1.5984	
-4.152	-1.5608	-0.93387	-2.3719	
-2.7258	-3.1213	0.0074522	-3.6727	
-1.2148	4.0949	0.13766	-4.5888	

Neural network weight and bias for Yield strength

IW		LW	B1	B2
-1.526	4.023	2.1164	4.553	-1.1197
-0.22131	4.5498	-0.38493	-3.5078	
0.28086	4.3132	-0.05222	-2.3219	
4.1682	-0.2297	1.7839	-2.5589	
4.2438	0.42192	-1.1408	-0.13543	
1.9429	-3.9596	0.12785	0.80051	
-2.4126	-3.3307	0.4491	-2.5417	
4.7969	0.31626	1.3294	1.873	
0.71493	-4.7666	-0.44633	2.8774	
-1.9091	4.2094	0.96401	-4.2162	

Neural network's weight and bias for Young Modulus

IW		LW	B1	B2
2.8004	-3.1176	0.36823	-4.6656	-0.71739
3.1357	3.0576	0.56977	-3.4973	
-3.0049	3.3767	1.0615	2.32	
4.4383	-0.22631	0.7899	-1.3553	
1.7816	4.0579	0.47505	-0.39927	
-3.3798	-2.8729	0.8244	-0.50857	
-4.0678	-1.7276	0.17344	-1.4977	
0.61389	4.3956	-0.54349	2.4711	
3.7662	2.0965	0.42818	3.5398	

3.8071	3.4052	0.82552	3.5591
